# Is femoral vein occlusion underestimated with extravascular hemostasis using biodegradable collagen plugs?

**DOI:** 10.1016/j.hrcr.2025.10.016

**Published:** 2025-10-21

**Authors:** Masataka Narita, Hitoshi Mori, Kazuhisa Matsumoto, Yoshifumi Ikeda, Ritsushi Kato

**Affiliations:** Department of Cardiology, Saitama Medical University, International Medical Center, Hidaka-city, Japan

**Keywords:** Catheter ablation, Extravascular hemostatic device, Femoral vein occlusion, Complication, Optimizing the use of the device

**Key Teaching Points**

▪ VASCADE MVP (Cardiva Medical) is an extravascular hemostatic device that uses a bioabsorbable collagen plug and has been reported to have a low complication rate, with proven efficacy in reducing postoperative bed rest time.
▪ In this case, asymptomatic right femoral vein occlusion was caused by an extravascular hemostasis device but resolved spontaneously with continued anticoagulant therapy without surgical intervention.
▪ Femoral vein stenosis or occlusion may be more common than previously recognized, and we considered it important to minimize the failure rate of temporary hemostasis by optimizing the use of the VASCADE MVP device.

## Introduction

VASCADE MVP (Cardiva Medical) is an extravascular hemostatic device that uses a bioabsorbable collagen plug and has been reported to have a low complication rate, with proven efficacy in reducing post-operative bed-rest time.^1^ A case has been reported of refractory deep vein thrombosis caused by a suture-mediated vascular closure device.^2^ We report a case of subclinical femoral vein occlusion caused by an extravascular hemostatic device using a bioabsorbable collagen plug.

## Case report

A 72-year-old woman was admitted to the hospital for catheter ablation of symptomatic paroxysmal atrial fibrillation. Four months before admission for catheter ablation, the patient developed diplopia due to left abducens nerve palsy and was referred to our neurosurgery department for further evaluation. On the same day, cerebral angiography revealed a dural arteriovenous fistula of the cavernous sinus, for which the patient underwent 2 endovascular treatments. During hospitalization, atrial fibrillation was detected, and apixaban 5 mg twice daily was initiated.

**Figure 1:**
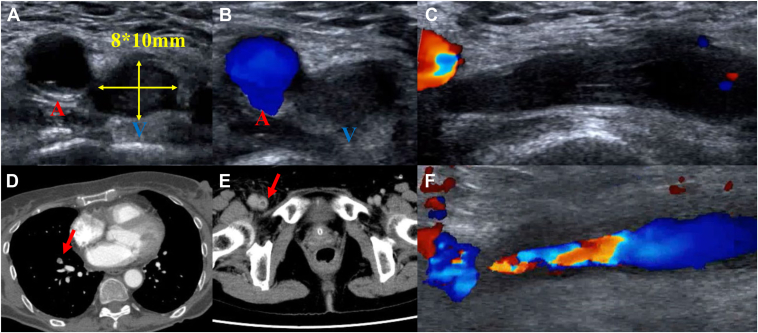
Vascular ultrasound in (**A–C**) demonstrates occlusion of the right femoral vein. The left vessel corresponds to the right femoral artery and the right vessel to the right femoral vein (**A–B**). Contrast-enhanced computed tomography revealed a contrast defect in a peripheral branch of the right lower pulmonary artery (**D**) and an occlusion of the right femoral vein (**E**). A follow-up lower limb venous ultrasound on the fourth hospital day demonstrated recanalization of the lower leg veins, although stenosis remained (**F**).

She was admitted for catheter ablation for atrial fibrillation. Anticoagulation was transitioned from apixaban to dabigatran, which was continued perioperatively for the catheter ablation procedure. A 7 Fr sheath, an 8 Fr sheath, and an 8.5 Fr VIZIGO sheath were inserted into the right femoral vein at 6 mm intervals in a caudal-to-cranial orientation. We created a 3-dimensional (3D) mapping of the left atrium using SOUNDFAM with CARTO (Biosense Webster) under intra-cardiac echocardiography guidance and performed an atrial septal puncture. Pulse applications were delivered to the left and right pulmonary veins using VARIPULSE (Biosense Webster). A residual potential was detected at the bottom of the right inferior pulmonary vein on the post-procedural map. Therefore, 2 additional pulses were delivered, followed by cavotricuspid isthmus ablation using radiofrequency ablation. The procedural duration was 2 hours and 9 minutes. During the procedure, the activated clotting time was consistently above 350 seconds, and the final activated clotting time was 408 seconds. Protamine 5 mL was administered, and hemostasis was attempted using 3 VASCADE MVP devices with adjustments in the angle and pressure to achieve temporary hemostasis. However, temporary hemostasis was not obtained, and oozing persisted despite sleeve withdrawal. The collagen plug was deployed 1 minute after hydration, and the 3 devices were subsequently removed. However, brisk backbleeding was observed, necessitating manual compression. As spontaneous hemostasis was not achieved after more than 20 minutes, a HemCon patch (HemCon Medical Technologies) was applied, followed by 40 minutes of compression to achieve hemostasis. Subsequently, a hemostatic pad and tape were used for compression, and the bandage was removed the next morning after 18 hours of compression. After compression was released, there were no symptoms, wound swelling, or vascular bruit. Because of difficulty in achieving hemostasis and the possibility of a vascular complication, lower limb vascular ultrasound was performed, revealing an occlusion of the right femoral vein at the puncture site (Figure 1A–1C, Supplemental Video 1A–1C). Blood tests showed no elevation in the D-dimer levels, which measured 0.49 μg/mL. However, contrast-enhanced computed tomography revealed an occlusion of the right femoral vein and a contrast defect in the peripheral branch of the right lower pulmonary artery (Figure 1D and 1E). At the time of admission, the patient was prescribed dabigatran 110 mg twice daily. Because venous thrombosis was suspected, anticoagulation therapy was switched to edoxaban 30 mg once daily. On the fourth hospital day, a lower limb venous ultrasound confirmed recanalization of the lower leg veins despite the presence of residual stenosis (Figure 1F, Supplement Video 1D). At that time, the lower limb circumferences measured 39 cm in the right thigh, 28 cm in the right lower leg, 38 cm in the left thigh, and 28 cm in the left lower leg, with no significant differences between the 2 sides. On the seventh hospital day, a lower leg venous ultrasound showed a further reduction of the plug. On the ninth hospital day, the anticoagulant was switched from edoxaban to apixaban 5 mg twice daily, which was the pre-admission dose, and the patient was discharged the same day. One month after discharge, a lower limb venous ultrasound confirmed no residual plug.

## Discussion

VASCADE MVP can achieve hemostasis at the puncture site without the use of contrast agents or imaging. However, complete hemostasis with VASCADE MVP may be challenging in some cases. In this case, femoral vein occlusion was considered to have resulted from failure of the VASCADE MVP to adequately seal the puncture site, leading to unsuccessful hemostasis and intravascular plug retention.

**Figure 2:**
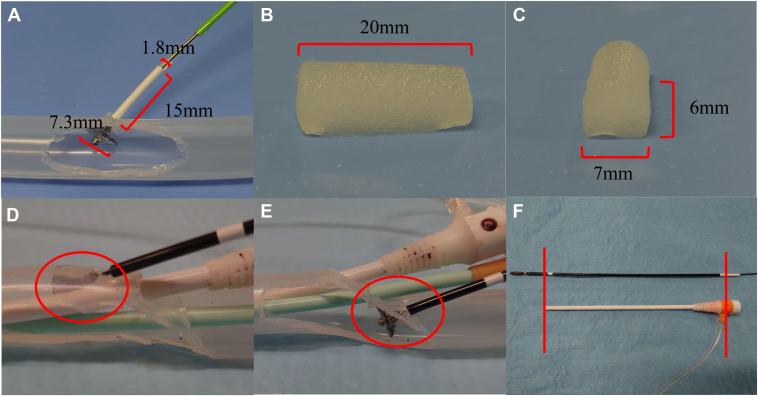
In an ex vivo experiment, a silicone hose with an inner diameter of 10 mm was used to simulate the femoral vein. (**A**) shows the expanded VASCADE MVP disk, illustrating the size of the collagen plug prior to hydration. The collagen plug was hydrated, after which its length, width, and height were recorded (**B, C**). **D:** shows the VASCADE MVP disk deployed from the cranial side of the sheath positioned in the femoral vein, whereas (**E**) shows deployment from the caudal side. As shown in (**F**), comparing the device length with the sheath is a useful method for confirming proper adherence of the VASCADE MVP disk to the vessel wall.

In ex vivo experiments, the femoral vein is simulated using a silicone hose with an inner diameter of 10 mm. The diameter of the VASCADE MVP disk measures 7.3 mm. Hemostasis is achieved at the puncture site with a disk, allowing the plug to be deployed outside the vessel (Figure 2A). After the collagen hydration, the plug measured 20 mm in length, 7 mm in width, and 6 mm in height (Figure 2B and 2C). If temporary hemostasis can be achieved with the VASCADE MVP disk, the likelihood of intravascular plug exposure is low. The potential causes of intravascular plug exposure include failure of temporary hemostasis due to interference from venous valves or the sheath, as well as excessive manual compression or the use of hemostatic pads during hemostasis. Assuming a femoral vein diameter of 10 mm, exposure of 2–3 plugs within the vessel could lead to venous narrowing or occlusion.

We evaluated the causes of temporary hemostasis failure in ex vivo experiments using VASCADE MVP with 3 sheaths. Deployment of the VASCADE MVP through the sheath positioned cranially in the femoral vein resulted in sheath interference, thereby hindering temporary hemostasis (Figure 2D). Sheath interference was thought to occur on the cranial side of the femoral vein due to a narrow angle between the vessel wall and the sheath, which caused the disk to become caught. In contrast, interference appeared to be reduced on the caudal side because of the wider angle (Figure 2E). Another approach that was considered was to evaluate the disk using vascular ultrasound; however, this proved difficult. As an alternative to ultrasound, Figure 2F shows that comparing device length with sheath length is a useful method to ensure that the VASCADE MVP disk adheres to the vessel wall. When expanding the disk, we also considered a method to prevent intravascular plug exposure by using the sheath length of the side port of the sheath and the white sleeve of the VASCADE MVP as guides. Because the length of the VASCADE MVP sleeve is not visible, comparing it with the sheath length can provide a reference point for the distance from the VASCADE MVP disk to the vessel wall. Other basic methods to facilitate temporary hemostasis include increasing the distance between puncture sites to at least 6 mm and adjusting the angle or orientation of the disk.

In this case report, femoral vein occlusion caused by plugs resolved spontaneously with continued anticoagulant therapy, suggesting that anticoagulation may help prevent asymptomatic femoral vein occlusion. Various strategies should be considered to prevent plug-induced femoral vein occlusion.

## Conclusion

In this case, asymptomatic right femoral vein occlusion was caused by an extravascular hemostasis device, but it resolved spontaneously with continued anticoagulant therapy. Femoral vein stenosis or occlusion may be more common than previously recognized, highlighting the importance of minimizing the failure rate of temporary hemostasis.

## Disclosures

HM received lecture fees from Biosense Webster Japan and Boston Scientific Japan. Our department received grant support from Boston Scientific Japan. All other authors have no other conflict of interest to disclose.
