# Supplementary figures and images for: Is femoral vein occlusion underestimated with extravascular hemostasis using biodegradable collagen plugs?
Source: HeartRhythm Case Rep. 2025 Oct 21;12(1):24–6. doi: 10.1016/j.hrcr.2025.10.016 (PMC12925722; doi:10.1016/j.hrcr.2025.10.016)

## Slide 1
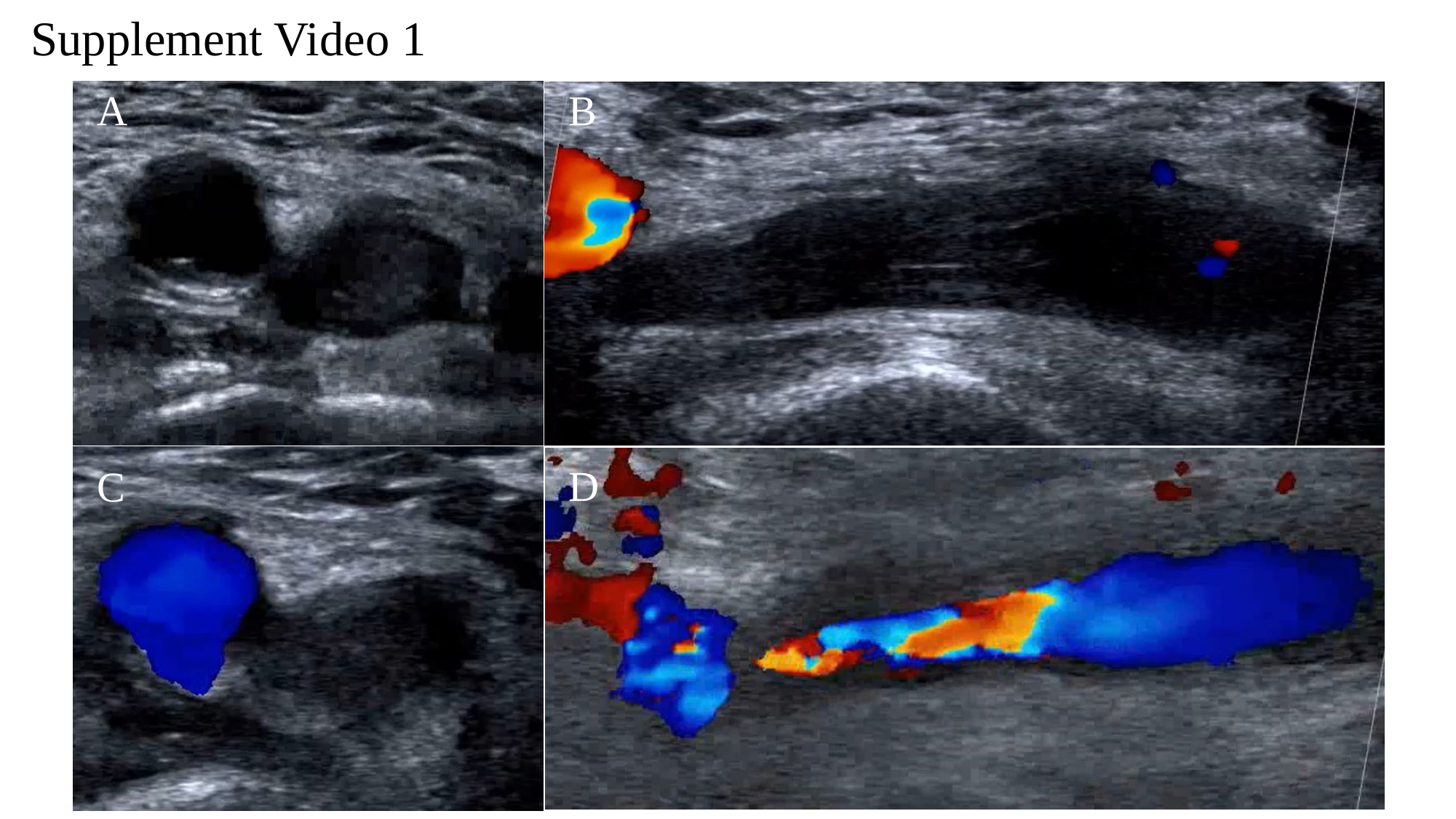

# Supplement Video 1
A
B
D
C

Supplement: Supplementary Video — 1 [file mmc1.pptx]
