# Supplementary material for: Is femoral vein occlusion underestimated with extravascular hemostasis using biodegradable collagen plugs?
Source: HeartRhythm Case Rep. 2025 Oct 21;12(1):24–6. doi: 10.1016/j.hrcr.2025.10.016 (PMC12925722; doi:10.1016/j.hrcr.2025.10.016)
Supplement: Supplementary Material [file mmc2.docx]

**Supplement Video 1.**

Supplementary Videos 1A–1C demonstrate right femoral vein occlusion on vascular ultrasound, while Video 1D demonstrates improvement on the fourth hospital day.
